# Interocularly merged face percepts eliminate binocular rivalry

**DOI:** 10.1038/s41598-017-08023-9

**Published:** 2017-08-08

**Authors:** P. Christiaan Klink, Daphne Boucherie, Damiaan Denys, Pieter R. Roelfsema, Matthew W. Self

**Affiliations:** 1 Vision & Cognition, Netherlands Institute for Neuroscience, KNAW, Amsterdam, The Netherlands; 2 Neuromodulation & Behavior, Netherlands Institute for Neuroscience, KNAW, Amsterdam, The Netherlands; 3 Department of Psychiatry, Academic Medical Center, University of Amsterdam, Amsterdam, The Netherlands; 4 Department of Integrative Neurophysiology, Centre for Neurogenomics and Cognitive Research, VU University, Amsterdam, The Netherlands

## Abstract

Faces are important visual objects for humans and other social animals. A complex network of specialized brain areas is involved in the recognition and interpretation of faces. This network needs to strike a balance between being sensitive enough to distinguish between different faces with similar features, and being tolerant of low-level visual changes so that a given face is stably perceived as a particular individual. Such stability may require feedback from higher brain regions down to the level where details are represented. Here, we describe a phenomenon in which interocular competition between face features is stabilized and eliminated when observers attend high-level face characteristics. Two different face images presented to the individual eyes do not cause the perceptual fluctuations that are typically observed in binocular rivalry. Instead, they merge into a stable percept of an intermediate face that combines features from both eyes’ images. The stability of the intermediate face percept depends on the observer attending holistic face properties such as identity or gender. It disappears when observers explicitly attend facial features, suggesting a crucial role of top-down stabilizing feedback from high-level areas that represent holistic faces back to lower processing levels where detailed face features compete for conscious representation.

## Introduction

**Figure 1:**
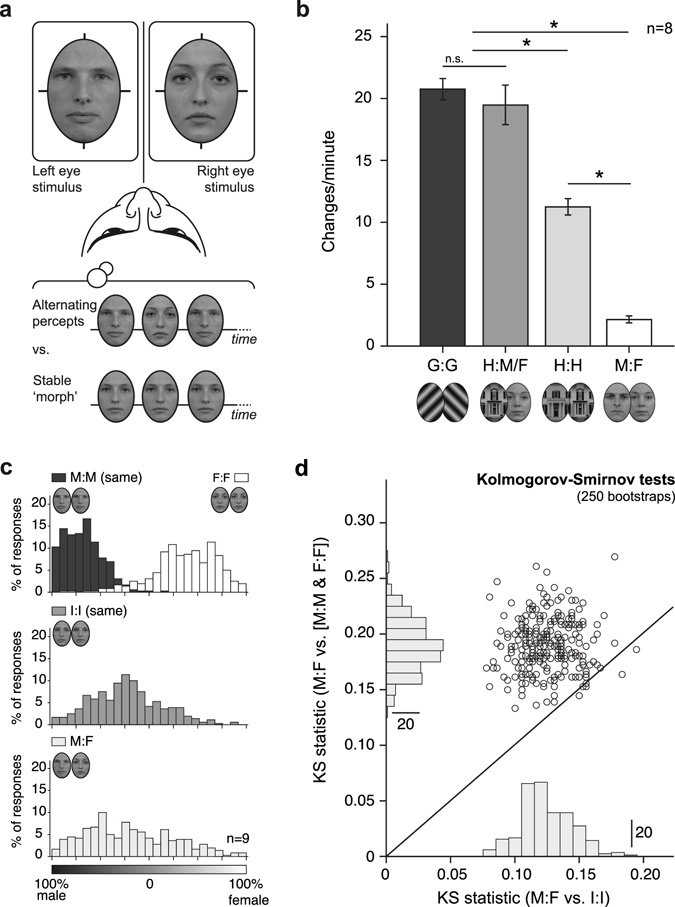
Conflicting face images interocularly combine into intermediate face percepts. (**a**) A male and female face image presented to the individual eyes do not evoke the perceptual alternations that are typical for binocular rivalry, but instead morph into a stable intermediate face percept. (**b**) Reported changes per minute for different continuous rivalry conditions. G:G denotes rivalry between two orthogonal gratings, H:M/F is rivalry between a house image and male or female face image, H:H is rivalry between house images, and M:F is a male and female face. Multiple comparisons (Tukey’s HSD) testing shows significant differences (*p < 0.001) between all conditions, except G:G and H:M/F, showing that faces hardly rival, but houses also rival less than stronger contrasting images. Error bars are ±1 SEM. (**c**) Gender rating distributions of Experiment 2. Individual eyes were presented with either two of the same male (M:M) or female (F:F) faces, a male and a female face (M:F), or two identical intermediate morph faces (I:I) and observers indicated their perceived gender on a continuous scale from fully male to fully female. (**d**) KS-statistics for 250 bootstrapped comparisons of the distributions in (**c**).

In binocular rivalry, two different images are presented to the individual eyes. This set-up typically leads to perceptual fluctuations between the two images every few seconds^1, 2^. Because of this clear dissociation between visual input and conscious visual experience, vision scientists often use binocular rivalry demonstrations to inform a general audience about the decisive role of the brain in visual perception. On one such an occasion, we attempted to demonstrate the binocular rivalry phenomenon with conflicting male and female face images and serendipitously noticed that, instead of the expected fluctuations between perceiving male and female faces, we perceived a stable intermediate, androgynous face (Fig. 1a). The fact that the stable face percept eliminated typical binocular rivalry alternations suggests a role of top-down stabilization mechanisms in face perception, whereas the intermediate androgynous nature of the percept implies that face feature information from both eyes is used to generate the percept. We set out to formalize this phenomenon and investigate its mechanism in a series of experiments.

It was later brought to our attention that interocular face merging had already been informally described in a letter to Charles Darwin, sent in 1877 and discussed in the works of Sir Francis Galton^3^. It was qualitatively explored about 80 years later^4^, yet despite this early exploratory study encouraging “further study to provide a satisfactory account of the effect”^4^, we believe that our current work offers the first controlled investigation of the phenomenon.

## Results

### Stable face percepts eliminate switches during prolonged binocular rivalry

In Experiment 1, we compared the perceptual dynamics of continuous binocular rivalry between two face images (M:F) with those of rivalry between gratings (G:G), house and face images (H:F), and two house images (H:H). Eight observers reported perceptual changes in two-minute rivalry trials (Fig. 1b). There were hardly any perceptual switches reported with the male and female face images, suggesting that the interocularly merged face percepts were indeed remarkably stable. Binocular rivalry between two house images also evoked significantly less perceptual switches than the more conventional rivalry between orthogonal gratings or house and face images but significantly more than between the two faces (one-way ANOVA with factor stimulus type F(3,28) = 77.8, *p* < 0.001; Tukey’s HSD multiple comparison’s tests, all *p* < 0.001, except G:G vs. H:F *p* = 0.80). This suggests that part of the stability of interocularly merged face percepts may be due to the high level of similarity between the two face images, but another important part is presumably due to mechanisms that are typical for face perception. We investigate this further in the remaining experiments.

### Interocular face combination yields intermediate face percepts

Experiment 2 addressed the apparent interocular perceptual merging of monocularly presented face images. We briefly (1,000 ms) presented observers with a male face image in one eye and a female face image in the other eye (M:F) and asked them to rate the gender of the face they perceived by setting a slider between 100% male and 100% female. In other conditions of this experiment we presented the two eyes with identical images of either male or female faces (M:M/F:F), or a morph image that we constructed to be exactly intermediate between male and female (I:I). The distributions of reported gender ratings were compared among stimulus conditions with the rationale that if male-female face image combinations rival, observers would either perceive the male or the female face and gender rating distributions would be bimodal and comparable to the combined distribution of the all-male and all-female conditions. If, however, male-female face combinations merge into a stable intermediate face, gender rating distributions are more likely to be unimodal, centered around the middle of the male-female scale and comparable to the intermediate morph condition. The latter is what we found (Fig. 1c). Gender rating distributions for combinations of male and female faces were unimodal (Hartigan’s dip test, D = 0.012, *p* = 1)^5^, as were those of intermediate morph images (D = 0.021, *p* = 0.40), but the combined distribution of all-male and all-female faces was bimodal (D = 0.059, *p* < 0.001). A direct comparison of these distributions using Kolmogorov-Smirnov tests indicated that the gender-rating distribution for male-female images was significantly distinguishable from both the same-gender (KS_[M:F vs. M:M/F:F]_ = 0.16, *p* < 0.001) and morph image distributions (KS_[M:F vs. I:I]_ = 0.11, *p* < 0.05). The higher KS statistic for M:F vs. M:M/F:F suggests that these gender-rating distributions are more dissimilar than those for M:F vs. I:I. To formally test this, we bootstrapped the Kolmogorov-Smirnov tests on gender ratings 250 times (Fig. 1d). One-tailed Mann-Whitney U tests on the distributions of KS statistics confirmed that gender ratings for male-female images were more similar to intermediate morph image distributions than to the combined male-male/female-female distributions (KS_[M:F vs. I:I]_ < KS_[M:F vs. M:M/F:F]_: *p* < 0.001, KS_[M:F vs. I:I]_ > KS_[M:F vs. M:M/F:F]_: *p* = 1).

### Attention to holistic faces stabilizes rivaling face features

In Experiment 3A, we investigated the interocular face merging phenomenon in a different way. We reasoned that if the intermediate face percept is indeed a composite of face feature information from both eyes, then removing the image from one eye during the stable percept should always cause a change in perceived face identity as some of the features which contributed to the mixed percept will disappear. If, however, the two face images would rival as in traditional binocular rivalry, then removing one of them would only cause a perceptual change if the removed image was perceptually dominant at the time of removal. In addition, to test whether the stabilization of face rivalry could be a specific consequence of the specialized face perception system we also performed the experiment with upside-down faces that are thought to engage the brain’s face perception system to only a limited extent^6–10^.

**Figure 2:**
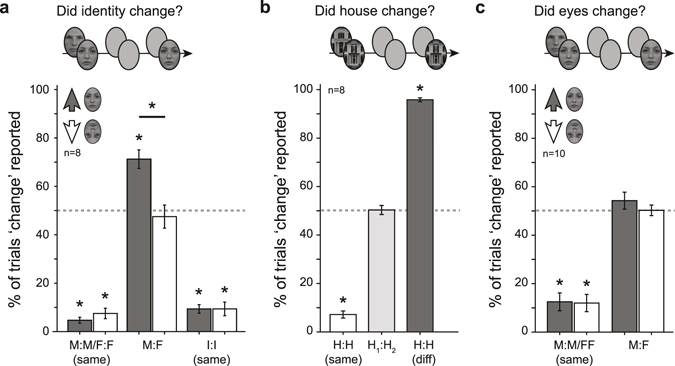
Interocular merging of face images depends on attention to holistic face properties. (**a**) Results from Experiment 3A. Percentage of trials in which observers reported an identity change between pre- and post-blank presentation intervals. M:M, F:F and I:I denote male, female and intermediate morph face images in both eyes respectively. For M:F there was a male face in one eye and a female face in the other eye. Gray bars depict results for upright faces, white bars upside-down faces (*p < 0.01, compared to of the level expected for rivalling stimuli of 50%). (**b**) Results from control Experiment 3C. Percentage of trials in which observers reported a change in perceived house between pre- and post-blank presentation intervals. For H:H conditions, the same house images were presented to both eyes and probed with either that same (‘same’, white) or a different (‘diff’, dark gray) image in the post-blank interval. For H_1_:H_2_, there were different house images presented to the individual eyes in the pre-blank interval and only one of the two images was presented again after the blank. (*p < 0.001, compared to a chance level of 50%). (**c**) In Experiment 4, observers reported eye changes for M:M and F:F face images (*p < 0.001, compared to a chance level of 50%). For M:F, they report eye changes on 50% of trials supporting the notion that interocular merging does not occur when features instead of holistic faces are attended. Error bars are ± SEM.

Face images were presented to the two eyes for 1,500 ms. After a brief blank interval (200 ms), only one of the images returned on the screen for another 1,500 ms and observers reported whether face identity changed across the blank interval. As expected, for all conditions with two identical images (M:M, F:F, or I:I) observers rarely reported a change (False positive rate = 7.2%) (Fig. 2a). For the upright male-female face condition, however, observers reported more identity changes than the expected rate of 50% for rivalling stimuli (one-tailed t-test, t(7) = 5.54, *p* < 0.001). This was a significantly higher rate than that observed for upside down faces (t(7) = 6.44, *p* < 0.001) for which the reported rate was indistinguishable from 50% (t(7) = −0.52, *p* = 0.69). Moreover, while changes from the interocular perceptual morph were on average reported at a slightly higher rate when probed with a male face (76% ± 5% SEM) compared to when probed with a female face (66% ± 4%)(two-tailed t-test, t(7) = 2.73, *p* < 0.03), both cases had a reported change rate that was significantly higher than 50% (one-tailed t-tests, both *p*’s < 0.01).

To ensure that these results were not due to difficulties in recognizing identity changes in upside-down faces, we carried out a control experiment (Experiment 3B) in which we repeated those conditions of Experiment 3 A in which the same images were presented to the two eyes in the first interval (i.e. M:M, F:F and I:I), but now in half of the trials we presented a different face image in the second interval, expecting this to lead to high levels of identity change detection. For M:M and F:F, changes were detected well above 50% (one-tailed t-tests, *p* < 0.01) and observers were equally accurate for upright and upside-down faces (t(7) = 1.08, *p* = 0.32). In all other conditions, performance was slightly better for upright than for upside-down faces (all *p* < 0.05), but both changes and no-changes were reported well above 50% for both orientations (all *p* < 0.01).

Since the continuous rivalry paradigm in Experiment 1 showed that rivalry between two similarly looking house images also evoked significantly less perceptual switching than rivalry between gratings or between houses and faces, we wondered whether the interocular merging we observed for face images would also to some extent be present for house images. To test this idea, we performed another control experiment (Experiment 3C). Here, we presented the same or different house images to the two eyes. After a brief blank interval, only one of the house images re-appeared and observers reported whether the houses they perceived before and after the blank were the same or different. The results were strikingly different from those with face stimuli in Experiment 3A (Fig. 2b). Changes were reported in the rivalry condition (i.e., two different houses presented before the blank) on approximately 50% of the trials (50% ± 2%; two-tailed t-test against 50%, t(7) = 0.17, *p* = 0.87). For individual observers, biases were observed towards one of the two houses dominating the rivalry (i.e., a high probability that observers reported a perceptual change when the other house image was shown after the blank). This effect was however not significant at the group level (change rate when probed with house 1: 65% ± 8%, t(7) = 1.86, *p* = 0.11; change rate when probed with house 2: 35% ± 6%, t(7) = −2.31, *p* = 0.05; house 1 vs. house 2: t(7) = 2.11, *p* = 0.07). In cases where the same images were shown to the two eyes before the blank, observers reliably reported both changes (96% ± 1%, t(7) = −29.11, *p* < 0.001) and repetitions (7% ± 1%, t(7) = 56.06, *p* < 0.001).

Since the face perception system is thought to process holistic faces and facial features with independent mechanisms^11–15^, we also investigated whether the interocular merging of face images is specific for observers attending holistic face features, like identity (Experiment 4). To this end we asked observers to focus on the eyes of the faces and report changes in this feature. Using the same paradigm as for Experiment 3A, observers were presented with either two identical face images (male or female), or with a male face image in one eye and a female face image in the other eye. Only one of the images was presented again after the blank interval and observers were asked whether they perceived any changes specifically in their perception of the eyes before and after the blank. In contrast to when subjects attended the identity of the face, the observers reported changes in the eye-percept on approximately 50% of trials. This proportion was independent of whether the second image was a male (48% ± 5%) or female face (54% ± 4%)(two-tailed t-test, t(18) = −0.89, *p* = 0.39), suggesting that face features indeed engaged in rivalry (Fig. 2c). The results were similar regardless of whether the faces were presented in an upright or inverted orientation (comparison to 50% level, upright: t(9) = 1.22, *p* = 0.26, upside-down: t(9) = 0.11, *p* = 0.91; upright vs. upside-down: t(9) = 0.87, *p* = 0.41). The results did not reflect an increased tendency to report eye changes as observers rarely reported a change when the post-blank face was identical to the pre-blank faces (12.0% and 12.5% for upright and upside-down faces respectively).

### Monocular grouping cues prevent interocular merging of face percepts

**Figure 3:**
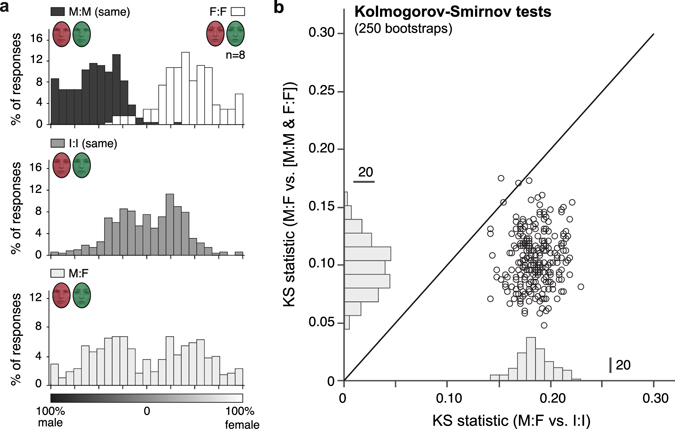
Monocular grouping cues prevent interocular merging of face images. (**a**) Gender rating distributions of Experiment 5. Individual eyes were presented with either two of the same male (M:M) or female (F:F) faces, a male and a female face (M:F), or two identical intermediate morph faces (I:I) overlaid with an eye-specific color (red or green). Observers indicated the perceived gender of each stimulus presentation on a continuous scale from fully male to fully female. (**b**) KS-statistics for 250 bootstrapped comparisons of the distributions in (**a**).

**Figure 4:**
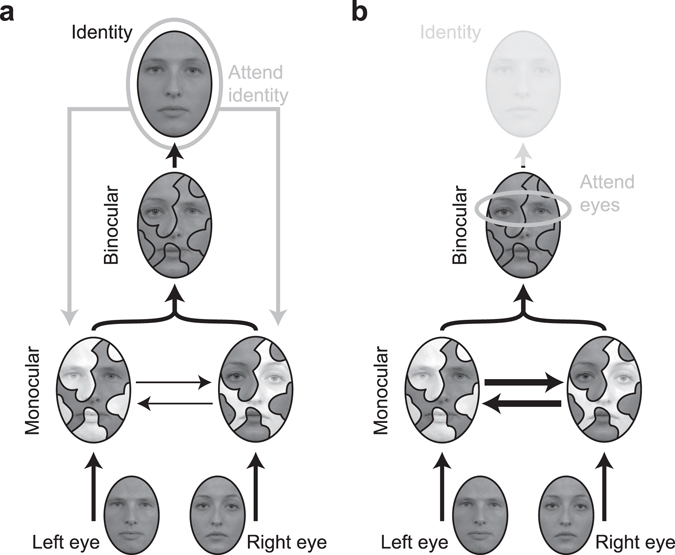
Potential mechanism behind the interocular face stabilization mechanism. (**a**) At a monocular, low level of processing, visual features presented to the individual eyes engage in binocular rivalry. The winning features in each eye (represented here as darker, high contrast areas) combine at a binocular level to form a full face percept. Once a coherent face has been established and higher-level processes have assigned it an identity, feedback back to the early rivalry process may stabilize the competition in favor of its constituent features (thin arrows). (**b**) When attention is directed to face features like eyes, instead of holistic aspects like identity, feedback does not occur and rivalry between face features ensues.

Our results thus far point to a mechanism in which face features may engage in binocular rivalry before being interocularly combined into an intermediate face percept that comprises visual information from both eyes. Once established, this face percept is then stabilized through top-down influences that rely on holistic face processing. We hypothesized that this mechanism would depend on a lack of monocular feature grouping cues and/or interocular feature contrast. To test this idea, we performed Experiment 5, a version of the gender-rating experiment (Experiment 2) in which we added color (red and green) to each eye’s image. The results confirmed our hypothesis by showing that the addition of monocular grouping cues (and color competition between the eyes) indeed eliminated the interocular establishment of androgynous intermediate face percepts (Fig. 3). While all gender rating distributions where bimodal (D_[M:M/F:F]_ = 0.056, *p* < 0.001, D_[M:F]_ = 0.407, *p* < 0.001, and D_[I:I]_ = 0.027, *p* < 0.05), a comparison of gender rating distributions (Fig. 4b) firmly established that gender ratings for male-female images were more similar to the combined male-male/female-female distributions than to intermediate morph image distributions (KS_[M:F vs. I:I]_ < KS_[M:F vs. M:M/F:F]_: *p* = 1, KS_[M:F vs. I:I]_ > KS_[M:F vs. M:M/F:F]_: *p* < 0.001).

## Discussion

Binocular rivalry is typically characterized as fluctuations between perceptual representations of conflicting stimuli presented to the two eyes. The perceptual fluctuations are however hardly ever this clear-cut and, depending on stimulus conditions, there can be a substantial proportion of the observation time when mixtures of the two stimuli are perceived instead^16–19^. The amount of interocular mixing and suppression can provide insight into the neural mechanisms of perceptual organization^18^. For conflicting face images, we have shown that in the absence of explicit monocular grouping cues or additional feature competition between the eyes, face information of the two eyes is integrated into a single percept.

Faces are a somewhat special class of visual objects, thought to engage a highly specialized neural machinery^11, 14, 15, 20^. An obligatory face detection stage has been suggested to gate access of visual information to this machinery based on an initial comparison of the incoming visual features against a rough but specific face template^20^. If the matching to this template depends on specific contrast values of the face and on its upright orientation, it would explain why many of the effects that distinguish face processing from object processing are absent if faces are displayed upside-down or with inverted contrast^6–9, 21^. In our experiments, the interocular merging of face information was absent when faces were presented upside-down, suggesting that this phenomenon specifically relies on properties of these specialized face processing modules.

The primate face perception system comprises a complex network of specialized brain areas^20, 22–27^. While different aspects of face perception have been shown to preferentially engage the left and right hemispheres^28–31^, recent findings from studies with monocularly presented images suggest that earlier cortical or even subcortical structures have specific roles in face perception as well^32–34^. In these studies, the detection of low-level differences between face images shown before and after a blank interval was better for face images that were monocularly shown to the same eye compared to images shown to different eyes. This advantage disappeared when observers were probed for more complex face features, like gender, implying that either the interpretation mechanisms that rely on holistic face percepts operate on binocular visual information only, or that estimating a gender difference does not require detailed low-level analysis of the individual face features. The interocular mixing of face features into holistic face percepts we observe in the current experiments suggests the former.

Our results have important implications for the understanding of binocular rivalry. The fact that participants perceived an ambiguous face composed of features originating from different eyes is difficult to explain as a result of global competition between the two eyes. Interocular combination of visual information into coherent percepts has previously been shown to occur in binocular rivalry^19^. However, in this previous study, interocularly grouped percepts were perceived only fleetingly and more recent studies have questioned whether these grouped percepts may have arisen due to a more local form of eye competition^35, 36^. Our results are consistent with a local form of eye competition in which a random selection of male and female monocular features win the competition for representation at binocular levels (Fig. 4). What is remarkable is that this interocularly grouped percept appeared almost completely immune to rivalry fluctuations. This result suggests that once the initial, local competition between the eyes establishes the chimeric face percept, this representation is somehow stabilized to prevent rivalry with a face composed of the non-selected features.

One likely source of this stabilizing factor is feedback from neurons representing the identity of the face. If these neurons actively select their preferred features and/or suppress their non-preferred features, then this may be sufficient to bias the competition between different features so that the identity of the face remains ‘locked-in’. This stability could be broken by specifically attending to one of the features (i.e., the eyes) of the face. In this case, the eyes appeared to rival as would be expected in traditional binocular rivalry. This result is consistent with the view that the stabilizing factor comes from a level higher than the processing of individual face features. This stabilization of face identity may be an important evolutionary adaptation allowing the face recognition system to be robust to changes in the incoming visual input (for example small changes in viewing angle or lighting conditions) and extract the unchanging identity of the face.

Is the stabilization of an interocularly merged percept specific for faces? Two dichoptically presented grating stimuli do generally not stabilize into a merged plaid percept, but we did observe that competing house images perceptually alternate at a lower rate than gratings or a house image competing with a face image. While this suggests that the amount of interocular conflict could play a role in the phenomenon, we found no evidence for the interocular merging of house images in the change detection paradigm. This could however be a matter of expertise. Faces are somewhat special visual objects because they are abundantly present, highly socially relevant, and stereotypical in their feature configuration. As a result, most humans are face perception experts and effortlessly assign high-level meaning to faces, like gender or emotion. It is conceivable that comparable expertise for another stimulus class could yield similar results, but it is difficult to imagine a stimulus class where such expertise exists. Perhaps expert house observers, like architects, will perceptually merge two dichoptically presented house images into an intermediate house percept that, to them, is clearly distinguishable from both constituent images on the basis of a holistic attribution that is inaccessible to non-experts. It would furthermore be interesting for future studies to investigate how two dichoptically presented face images are perceived by observers that lack the common holistic face perception expertise, for instance those that are diagnosed with prosopagnosia or autism spectrum disorder^37–39^.

## Methods

### Participants

Observers (Exp 1: n = 8; Exp 2: n = 9; Exp 3 A,B,C: n = 8; Exp 4: n = 10; Exp 5: n = 8) had normal or corrected-to-normal visual acuity and ranged in age between 20 and 37 years. They gave informed consent and all, except one author, were naive with respect to the aims of the study. All experimental procedures were approved by the Ethics Committee from the Faculty of Social and Behavioural Sciences at the University of Amsterdam and performed in accordance with all relevant guidelines and regulations.

### Stimuli

All stimuli were generated and displayed using the PsychToolbox extensions^40, 41^ in Matlab R2010a (The MathWorks Inc., Natick, MA) running under Microsoft Windows 7. They were presented on a gamma-corrected DELL Trinitron 21″ CRT Monitor (1280 × 1024, 85 Hz) and viewed through a mirror stereoscope with a chinrest at a distance of 51 cm. To facilitate binocular fusion, all rivalry images were 3 degrees wide, 4 degrees high and drawn inside a 3.5 by 4.5 degrees alignment frame, on top of an alignment cross with a horizontal section of 5 degrees and a vertical section of 6 degrees (Fig. 1a). To further aid fusion, 100 circles with a random size between 0.5 and 2 degrees and a random luminance between 30 and 70% of the screen’s white intensity, were randomly positioned on the neutral gray background.

Ten male (M) and ten female (F) face images, Caucasian, with frontal viewpoints, forward gaze, and neutral expressions, were selected from the Radboud Faces Database (RaFD)^42^. These images were cropped within an elliptical aperture, converted to grayscale, matched for contrast, and facial features were aligned. The house images and gratings (oriented at +45 and −45 degrees) of Experiment 1 were treated similarly. For ten pairs of unique male and female faces we used the Morph Age Pro (Creaceed, Mons) software to create a series of morph images between the male and female face based on 5 fiducial markers. The middle image of each series was selected as the ‘Intermediate morph face’ (I) of the two original images.

### Procedures

In Experiment 1, pairs of images (Grating:Grating, House:M, House:F, M:F, House:House) were shown for two minutes while observers reported any perceptual change by pressing a key on the keyboard. Each condition was shown twice, in random order and counterbalanced over the eyes. We analysed the rate of reported perceptual changes per condition.

In Experiment 2, pairs of face images were shown to the two eyes (M:M same, F:F same, M:F, I:I same) for 1,000 ms after which a slider appeared on the screen and participants used the keyboard to indicate how they judged the gender of their face percept by adjusting the slider between 100% male and 100% female. A total of 240 trials were performed per observer, equally spread over conditions, presented in random order, and counterbalanced over the eyes. We calculated the distributions of gender estimates across conditions. Unimodality of these distributions was evaluated with Hartigan’s Dip Test^5^. We determined whether gender-rating distributions were statistically distinguishable using Kolmogorov-Smirnov tests and estimated whether pairs of distributions differed in their similarity by bootstrapping Kolmogorov-Smirnov tests and comparing the resulting distributions of KS statistics with Mann-Whitney U tests.

In Experiment 3A, pairs of face images were shown to the individual eyes (M:M same, F:F same, M:F, I:I same) in upright or upside-down orientation for 1,500 ms. After a 200 ms blank interval during which only the alignment elements were displayed, only one of the original face images returned on the screen for another 1,500 ms and observers indicated whether they perceived a change of face identity across the blank by pressing one of two buttons on a keyboard. A total of 320 trials were performed per observer, equally spread over conditions, presented in a random order, and counterbalanced over the eyes. The probability of a reported change was evaluated with t-tests against the 50% level or between conditions.

In Experiment 3B, pairs of images (M:M, F:F, or I:I) were again shown for 1,500 ms followed by a blank 200 ms blank interval. In the 1,500 ms after the blank either one of the original images or a new image from the same category (M, F, or I) was shown to one of the eyes and observers reported whether they perceived an identity change by pressing one of two buttons on a keyboard. Both upright and upside-down faces were tested. A total of 160 trials were performed per observer, equally spread over conditions, presented in random order, and counterbalanced over the eyes.

Experiment 3C was similar to 3A and 3B, but this time pairs of either the same or different house images were shown to the individual eyes for 1,500 ms. After a blank period of 200 ms blank interval, only one of the original house images was shown to one of the eyes and observers reported whether this house was the same as, or different from, the house they perceived before the blank. 240 trials were performed per observer, equally spread over conditions, presented in random order, and counterbalanced over the eyes.

Experiment 4 replicated Experiment 3A with a subset of image pairs (M:M, F:F, M:F). Both images (either upright of upside-down) were again shown for 1,500 ms, followed by a 200 ms blank, and a 1,500 ms interval in which one of the images was shown again. Observers this time reported whether they detected a difference in the eyes before and after the blank by pressing one of two buttons on a keyboard. A total of 160 trials were performed per observer, equally spread over conditions, presented in random order, and counterbalanced over the eyes.

Experiment 5 replicated Experiment 2 with the only difference that each eye’s image was displayed in a different color, red. RGB intensities of 100%, 50% and 50% (red) and 32.5%, 32.5% 65% (green) were chosen based on subjective determination of being balanced during rivalry. The color-to-eye assignment was counterbalanced and pseudorandom.

### Data availability

Both the datasets generated during and/or analyzed during the current study and the experiment code are available from the corresponding author on reasonable request.
